# Hard Tissue Volume Stability Effect beyond the Bony Envelope of a Three-Dimensional Preformed Titanium Mesh with Two Different Collagen Barrier Membranes on Peri-Implant Dehiscence Defects in the Anterior Maxilla: A Randomized Clinical Trial

**DOI:** 10.3390/ma14195618

**Published:** 2021-09-27

**Authors:** So-Ra Lee, Tae-Sik Jang, Chang-Su Seo, In-Oh Choi, Won-Pyo Lee

**Affiliations:** 1 Department of Periodontology, School of Dentistry, Chosun University, Gwangju 61452, Korea; click7980@gmail.com (S.-R.L.); inono10@gmail.com (I.-O.C.); 2 Department of Materials Science and Engineering, Chosun University, Gwangju 61452, Korea; tsjang@chosun.ac.kr; 3 Department of Dental Materials, School of Dentistry, Chosun University, Gwangju 61452, Korea; ju10003o@chosun.ac.kr

**Keywords:** bone regeneration, clinical study, dental implants, tissue pressure, titanium

## Abstract

This single-blinded, randomized, controlled study aimed to clinically and radiographically evaluate hard tissue volume stability beyond the bony envelope using three-dimensional preformed titanium mesh (3D-PFTM) for peri-implant dehiscence defects in the anterior maxilla. A total of 28 patients who wished to undergo implant surgery combined with guided bone regeneration (GBR) after extraction of a single maxillary anterior tooth were randomly assigned to two groups depending on the type of collagen membrane used, additionally with the 3D-PFTM—test (n = 14, cross-linked collagen membrane; CCM) and control (n = 14, non-cross-linked collagen membrane; NCCM) groups. Each implant was evaluated radiographically using CBCT at baseline, immediately after surgery, and at 6 months postoperatively. The relative position and distances from the bony envelope to the outlines of the augmented ridge were further determined immediately after GBR and 6 months after healing. At the platform level, the mean horizontal hard tissue gain (HG) at all the sites was 2.35 ± 0.68 mm at 6 months postoperatively. The mean HG rate was 84.25% ± 14.19% in the CCM group and 82.56% ± 13.04% in the NCCM group, but the difference was not significant between the groups. In all cases, HG was maintained beyond the bony envelope even after 6 months of GBR. This study suggests that 3D-PFTM should be considered a valuable option for GBR for peri-implant dehiscence defects in the anterior maxilla. In addition, 3D-PFTM may confer predictable hard tissue volume stability even after the healing period of hard tissue augmented outside the bony envelope by GBR.

## 1. Introduction

Following tooth extraction or tooth loss, healing of the alveolar bone inevitably involves bone resorption. Securing a sufficient amount of alveolar bone is key to placing a long-term esthetically and functionally good implant on the alveolar ridge where alveolar bone loss has occurred [1,2]. Various methods, such as autologous onlay block bone graft, sinus floor elevation, rigid splitting, and guided bone regeneration (GBR), have been used to reconstruct alveolar bone loss. Among them, GBR is a method that has shown predictive bone regeneration results in many studies and is the most commonly used method, simultaneously with or before implant placement, to reduce fixture exposure [3,4,5,6,7].

In particular, since the labial bone is thin in the maxillary anterior region, the frequency of bone dehiscence and esthetic problems is higher than that in the posterior region due to physiological bone resorption after tooth extraction. Hammerle et al. [8] reported that if the buccal bone in the anterior maxilla is thin, additional GBR is required even if the bone is not damaged. However, the results of GBR in the maxillary anterior region are limited, depending on the materials and procedures used because of the thin labia and gingiva of the adjacent teeth, as well as the compression of the lips and tongue [9]. Specifically, when using only particulate bone and resorbable collagen membranes, which are most commonly used in the GBR process, compromised regeneration may occur, depending on the shape of the bony defect [10]. Due to their poor mechanical properties and low resistance to tissue collapse [11], compressive forces cause collapse of the membrane and downward displacement of the grafting material immediately after flap closure [12] or during the healing stage [13,14]. Jiang et al. [14] reported that when GBR was performed with particulate deproteinized bovine bone mineral (DBBM) and a resorbable collagen membrane at the same time as implant placement in the maxillary anterior region, continuous horizontal volume loss occurred during the healing period, and new bone formation in the shoulder area of the fixture could be predicted only within the bony envelope.

In our recent retrospective clinical study of 129 implant sites in 100 patients, when GBR was performed using a three-dimensional preformed titanium mesh (3D-PFTM) in the peri-implant non-contained defects, sufficiently predictable hard tissue gain was obtained [15].

Therefore, in this prospective, randomized controlled clinical study, the GBR effect of 3D-PFTM and two types of collagen membranes were evaluated clinically and radiologically in peri-implant dehiscence defects in a single loss of the maxillary anterior region. Furthermore, the stability of hard tissue volume overadded beyond the bony envelope using particulate bone grafts and 3D-PFTM was assessed during the healing period.

## 2. Materials and Methods

### 2.1. Study Design

This study was a prospective, single-blinded, randomized controlled clinical trial. The study protocol was approved by the Institutional Review Board (IRB) of Chosun University Dental Hospital (approval number: CUDHIRB-1705-001). All registered participants provided written informed consent in accordance with the IRB guidelines, and the study was conducted in accordance with the Declaration of Helsinki and the Guidelines on Good Clinical Practice.

### 2.2. Study Population

A total of 30 participants who wanted a single implant surgery after extraction of a maxillary anterior tooth were recruited from the Department of Periodontology at Chosun University Dental Hospital from October 2017 to December 2019. The inclusion criteria were as follows: (a) age ≥18 years; (b) single maxillary anterior tooth missing (#13–23); (c) presence of healthy periodontal adjacent teeth; (d) indication of implant placement with simultaneous GBR; and (e) healthy or well-controlled systemic conditions for implant surgery and GBR. The exclusion criteria were as follows: (a) indication of staged GBR due to severely atrophic alveolar ridges, (b) current smokers (>10 cigarettes/day), (c) bone metabolic disease, (d) uncontrolled periodontal disease, and (e) other systemic diseases or general health conditions, which would be a contraindication to implant surgery and GBR.

### 2.3. Randomization and Surgical Procedure

A total of 30 patients were randomized in a 1:1 ratio to the test (n = 15; cross-linked collagen membrane, CCM) and control (n = 15, non-cross-linked collagen membrane; NCCM) groups, using the Sealed Envelope database (https://www.sealedenvelope.com, accessed 16 August 2021) with stratified block randomization (fixed block sizes of 2 and 4) given by the independent allocator (C.-S.S.). Randomization was performed at the second visit of each enrolled patient (baseline phase), and the allocator hid the block size until completion of the clinical trial. The operator (W.-P.L.) was aware of whether the patient was assigned to the test or control group after opening the envelope at the time of the first stage of implant surgery.

**Figure 1:**
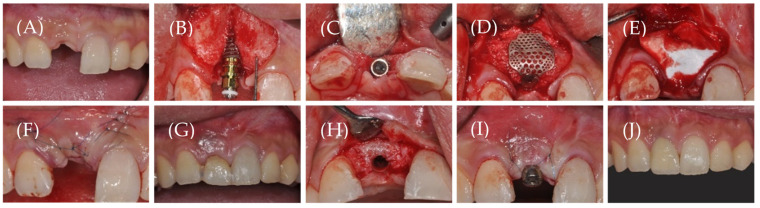
Surgical procedure stages. (**A**) Baseline: #11 is missing, (**B**,**C**) Implantation: peri-implant dehiscence bony defect is seen, (**D**) Bone grafts and 3D preformed titanium mesh application, (**E**) Collagen membrane application, (**F**) Suturing, (**G**) Healing state before second implant surgery, (**H**) Minimal incision and flap elevation: good hard tissue gain is noted, (**I**) Healing abutment installation and suturing, and (**J**) Final prosthetic restoration.

All surgical procedures were performed by a single skilled periodontologist (W.-P.L.). Gargling was performed for 1 min using a 0.12% chlorhexidine solution before surgery. The surgical area was anesthetized with 2% lidocaine containing epinephrine (1:100,000) by local infiltration (Figure 1A). Subsequently, bone exposure was facilitated by elevating a full-thickness flap after a mid-crestal horizontal incision and vertical incision with a #15 surgical blade. Following curettage of the inflamed tissue, cortical bone perforation was performed using a #330 carbide burr for bone grafts. Three types of implants (TS III SA^®^, Osstem, Seoul, Korea; Superline^®^, Dentium, Seoul, Korea; and Megagen, Daegu, Korea) were placed according to the standard guidelines of each manufacturing company. In all cases, the fixture thread was partially exposed to the buccal area (Figure 1B,C), and the GBR procedure was performed on the peri-implant bony defects. DBBM (Bio-Oss^®^, Geistlich Pharma AG, Wolhusen, Switzerland) and liquid platelet-rich fibrin were mixed and grafted onto the peri-implant dehiscence defects after fastening a 1-mm anchor screw (Osstem, Seoul, Korea) to the fixture. Subsequently, 3D-PFTM (Oss-builder^®^, Osstem, Seoul, Korea) was applied, and the mesh was fixed using a 0.3 mm-height cover screw (Osstem, Seoul, Korea) (Figure 1D).

CCM (Ossix Plus^®^, Datum Dental Biotech, Lod, Israel) was used in the test group, and NCCM (Jason membrane^®^, Botiss biomaterials GmbH, Zossen, Germany) in the control group (Figure 1E).

Subsequently, a periosteal-releasing incision was made to form a tensionless flap, and a primary suture was performed using a non-resorbable monofilament (Rexlon 5–0^®^, Metavision Co., Seoul, Korea) (Figure 1F). Immediately upon completion of surgery, cone-beam computed tomography (CBCT; CB MercuRay TM; Hitachi, Tokyo, Japan) was performed, and antibiotics (Augmentation 625 mg, Ilsung Pharm Co., Seoul, Korea) and analgesics (Aceclofenac 100 mg, Dona-A ST, Seoul, Korea) were administered orally for 7 days. Additionally, patients were taught to rinse the oral cavity with 0.12% chlorohexidine, which was done twice a day for 2 weeks. Complete stitch-out was performed 2 weeks after the surgery.

After a healing period of approximately 6 months, CBCT was performed again to evaluate changes in bone augmentation just before the second-stage implant surgery, and a second surgery was performed to remove the 3D-PFTM and mount the healing abutment (Figure 1G). After minimal incision for mesh removal, the full-thickness flap was elevated, and the cover and anchor screws used for fixing the 3D-PFTM were removed together with the mesh (Figure 1H). A healing abutment of an appropriate diameter and height was fastened and sutured (Figure 1I). Subsequently, analgesics (aceclofenac 100 mg, Dona-A ST, Seoul, Korea) were administered orally for 5 days. Prosthetic restoration was performed within 2–3 months of the second-stage implant surgery, and all patients were followed up for at least 6 months after the final restoration (Figure 1J).

### 2.4. Results Analysis

#### 2.4.1. Clinical Evaluation

Complications such as infection, edema, and exposure to 3D-PFTM were evaluated at 1 week, 2 weeks, 1 month, 3 months, and 6 months after the primary implant surgery. At the second stage of implant surgery, the implant stability quotient (ISQ) was measured for all implants.

#### 2.4.2. Radiological Evaluation

**Figure 2:**
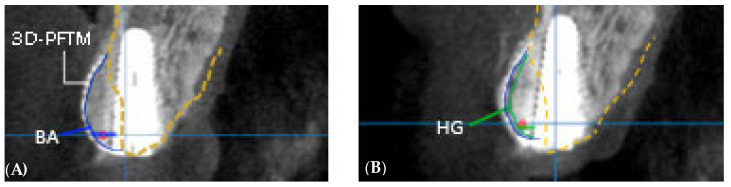
Method of radiographic analysis. A line parallel to the long major axis of the implant and a perpendicular line extending buccally at the level of the implant platform are drawn. (**A**) BA, bone augmentation immediately after surgery; (**B**) HG, hard tissue gain after 6 months of healing. The gap between the blue and green lines indicates bone resorption, while the red spot indicates the bony envelope. 3D-PFTM, three-dimensional preformed titanium mesh.

**Figure 3:**
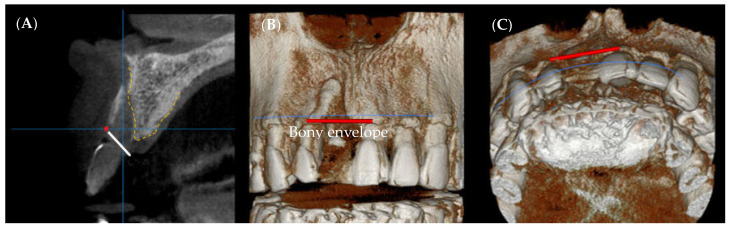
**(A)** CBCT sectional view. Note that the bony envelope appears as a red spot, (**B**,**C**) 3D radiographic analysis of bony envelope level.

For radiological evaluation, CBCT was performed for all implants under the same imaging conditions (FOV diameter, 10 cm; FOV height, 5.6 cm; acceleration voltage, 90 kV; beam currency, 8.0 mA; voxel size, 0.2 mm) preoperatively as well as immediately and 6 months (just before the second surgery) after the first surgery. Radiographic measurements were performed by a single independent investigator (S.-R.L.) who was uninvolved in the surgery and was blinded to the group allocation. In the measurement program (OnDemand 3-DTM, Cybermed, Seoul, Korea), cross-sectional computed tomography (CT) images across the center of the implant were used to compare the amount of bone regeneration obtained from the bony defects, and the line perpendicular to the long axis of the implant was extended from the platform level to the buccal side to measure the distance from the outermost bone on the extension line (Figure 2). The amount of bone graft reduction (BR) was defined as the amount of bone augmentation (BA) measured immediately after the first surgery minus the amount of new hard tissue gain (HG) after 6 months, and hard tissue gain rate (HGR) was defined as the percentage of HG compared with BA [15]. The position of the bony envelope was determined by the line connecting the most labial alveolar bone of both residual teeth in the 3D image (Figure 3), based on which the outside of the bony envelope was set as a negative value and the inside of the bony envelope as a positive value [14]. The length of the implant was measured, and measurement errors were corrected based on the magnification of the image in proportion to the actual length of the implant [15]. To improve the reliability of radiographic evaluation, the ‘test-retest reliability’ method was used. Five randomly selected CT radiographs were measured twice with a 4 week interval between measurements to assess intra-examiner reliability. Their relationships were compared using the Pearson correlation coefficient (r), and a reliable repeatability frequency (r > 0.90) was calculated. After that, all measurements were performed thrice and the average value was used.

### 2.5. Statistical Analysis

For the statistical analysis, 14 cases from each group, except for two with mesh exposures, were analyzed. Each quantitative variable was expressed as the mean and standard deviation. Both the test and control groups were tested for normality using the Shapiro–Wilk test. In addition, the Mann–Whitney test was used to check whether there was a significant difference between the groups. The significance level was set at *p* < 0.05. Statistical analysis was performed using SPSS version 22.0, for Windows (SPSS Inc., Chicago, IL, USA). The methodology was reviewed by an independent statistician.

## 3. Results

### 3.1. Characteristics of Participants

**Figure 4:**
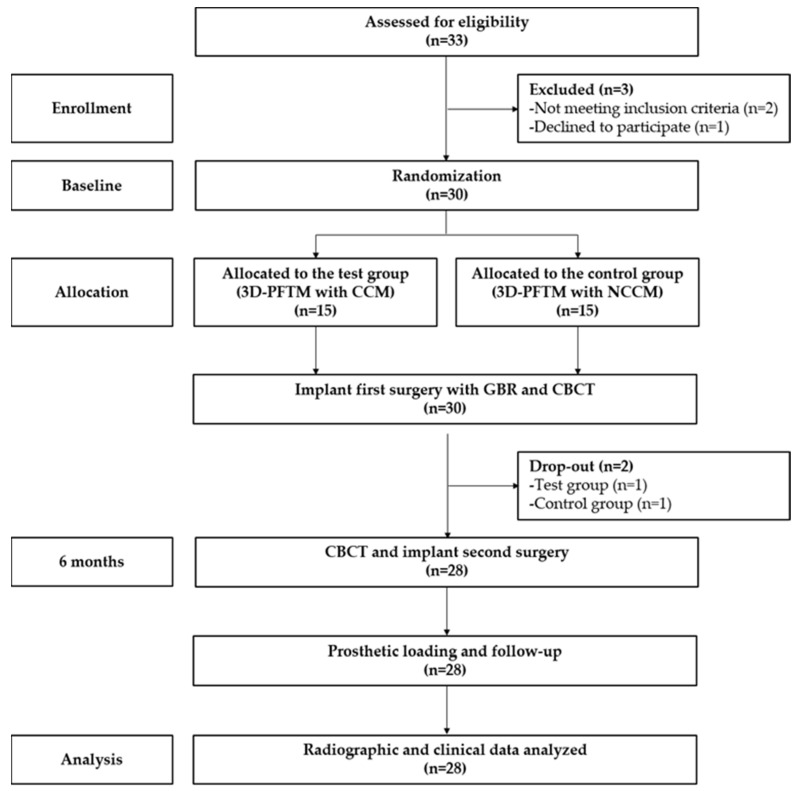
Flowchart for clinical trial enrollment. 3D-PFTM, three-dimensional preformed titanium mesh; CCM, cross-linked collagen membrane; NCCM, non-cross-linked collagen membrane.

A total of 30 participants were included, based on the inclusion and exclusion criteria. After GBR was performed at the same time as the first-stage implant surgery, one patient in the test group and one in the control group were excluded from 3D-PFTM exposure during the healing period. Finally, clinical and radiological evaluations were performed on 28 patients (14 in the test group and 14 in the control group) (Figure 4), and the overall average age was 48.9 years (range, 20–84 years) (Table 1).

### 3.2. Clinical Evaluation Results

The clinical evaluation results are summarized in Table 2. The mesh exposure rate for 30 patients recruited at the start of the study was 6.67% (2/30 patients; one patient in each group). In both groups, the average ISQ value was stable at >70, and the survival rate was 100%. During the surgery and follow-up, there were no unusual events or serious complications, except for general minor edema after surgery.

### 3.3. Radiological Evaluation Results

The radiological evaluation results are presented in Table 3. The total amount of BA was 2.83 ± 0.68 mm, with 2.91 ± 0.75 mm in the test group and 2.76 ± 0.62 mm in the control group; there was no statistically significant difference between the groups. After 6 months, the amount of HG and BR was 2.44 ± 0.75 mm and 0.47 ± 0.47 mm, respectively, in the test group and 2.27 ± 0.63 mm and 0.49 ± 0.40 mm, respectively, in the control group, indicating that there was no statistically significant difference between the groups. The total amount of HG and BR was 2.35 ± 0.68 mm and 0.48 ± 0.43 mm, respectively. The total HGR was 83.41% ± 13.40% and 84.25% ± 14.19% in the test group and 82.56% ± 13.04% in the control group, and there was no statistically significant difference between the groups.

**Figure 5:**
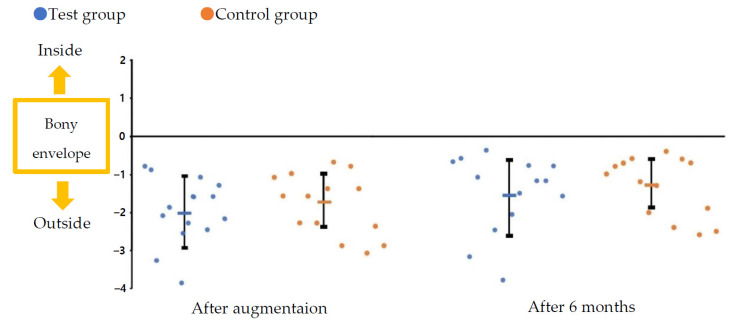
Distance from the boundary line of the bony envelope to the hard tissue profile immediately after augmentation and after 6 months of healing.

Immediately after GBR and 6 months after surgery, the distance from the boundary line of the bony envelope to the hard tissue profile was measured in the labial direction of the platform level of the implant fixture. Immediately following GBR, it was −2.01 ± 0.89 mm in the test group and −1.82 ± 0.82 mm in the control group, with an average of −1.91 ± 0.84 mm. Sufficient bone grafts were observed outside the bony envelope due to the overaddition of bone-substitute materials. Further, 6 months after surgery, the distance was −1.54 ± 1.02 mm in the test group and −1.34 ± 0.79 mm in the control group, with an average of −1.44 ± 0.90 mm. In all cases, HG was maintained outside the bony envelope in the anterior region, even after 6 months of GBR. All results showed no significant differences between the groups (Table 4 and Figure 5).

## 4. Discussion

In this study, the clinical and radiographic results of GBR using 3D-PFTM and two resorbable collagen membranes (crosslinked or non-crosslinked) were evaluated to treat dehiscence defects around the implants at the same time as implant placement in the maxillary anterior region. Additionally, after setting the bony envelope, we further evaluated whether the increased HG was stably maintained even after the 6 month healing period.

Conventional Ti meshes have excellent space maintenance capacity, which provides predictable results when performing extensive bone grafting, and they are less susceptible to bacterial infection. In addition, they allow a sufficient supply of blood, nutrients, and oxygen because of their macropore size, which promotes bone and soft tissue regeneration [16]. However, conventional Ti meshes are difficult to use as they require complete fixation at the appropriate position and must be cut and bent to the appropriate size and shape prior to application. Therefore, very high technical requirements are required, which leads to a high exposure frequency of the Ti mesh [17]. According to Rakhmatia et al., the conventional Ti mesh exposure rate ranges from 14.28% to 52.7% [18]. Previous studies using 3D-PFTM have reported high mesh exposure rates of 20%–25%, which are similar to those reported for the conventional Ti mesh [7,19]. Exposed meshes do not always lead to bone regeneration failure, but may exhibit lower implant success rates due to contamination and subsequent inflammation [7,16,20,21,22,23,24,25]. Nevertheless, in this study on a single site of the maxillary anterior region, exposure to 3D-PFTM was observed in two of the 30 patients initially recruited, resulting in an exposure rate of 6.67%. This was less exposure compared to that reported in the study by Funato et al. [26], in which the conventional Ti mesh showed an exposure rate of 20% in the maxillary anterior region. Unlike the conventional Ti mesh, 3D-PFTM does not need to be cut or bent and does not have a sharp margin; therefore, it can reduce surgery time and trauma to the flap, thereby reducing mesh exposure [7].

Some studies have recommended the additional use of a resorbable collagen membrane on the Ti mesh to compensate for the limitations of the conventional Ti-mesh [27,28,29]. A study by Lim et al. [29] reported that the exposure rate was not significantly reduced even with the additional use of a resorbable collagen membrane, but Strietzel et al. [30] reported that when a collagen membrane was used during GBR, the fibrous tissue grew within the collagen membrane and functioned as a barrier for space maintenance to prevent flap dehiscence. In our previous study, where only 3D-PFTM was used, an exposure rate of 11.8% was observed, whereas when CCM and NCCM were additionally used together, the exposure rate was reduced to 4.2% and 5.0%, respectively [15]. In this study, when CCM and NCCM were additionally used together with 3D-PFTM, the exposure rate was 6.67%, showing a slightly increased exposure rate compared to that reported in a previous study. In other words, 3D-PFTM exposure remains an issue in this type of surgery. Our previous study included not only the anterior region but also the posterior region, and since several implantation sites were mainly evaluated, the exposure rate may have increased in this study, which was performed only on the maxillary anterior and single regions. Furthermore, in a study related to GBR using a Ti-reinforced d-PTFE membrane in a single loss of the maxillary anterior region, Herzberg et al. [25] reported the necessity of caution as the exposure rate of non-resorbable membranes is relatively higher in a single tooth loss area than in a multiple tooth loss area.

The average horizontal bone loss after implant placement is 1.3–1.4 mm [31,32]. According to Simion et al. [33], marginal bone loss of approximately 2.11 mm during 1 year of functional loading is normal due to physiological phenomena. This phenomenon causes overall soft tissue loss and gingival recession, resulting in esthetic problems in the labial area. Therefore, in the anterior region where esthetics is important, rather than proceeding with implant placement at the same time as bone grafting, performing it in stages can yield much better results in terms of esthetics [25]. In general, implants with thick buccal bone can have long-term esthetic results, and the thickness of the buccal bone should be at least 2 mm [31]. In the case of the maxillary anterior region, it is generally difficult to predict augmentation due to compression of the lip muscles and soft tissues [25]. Therefore, it is advisable to perform excessively large horizontal bone grafts with overaddition of 2–4 mm or more to obtain long-term stability by supplementing the natural bone remodeling that appears around the implant [2,31,34,35]. In this study limited to the maxillary anterior region, the mean amount of BA immediately after GBR using 3D-PFTM was 2.83 ± 0.68 mm, and the mean amount of HG was 2.35 ± 0.68 mm, 6 months after surgery. There was no statistically significant difference in the amount of HG between the CCM and NCCM groups (2.44 ± 0.75 mm vs. 2.27 ± 0.63 mm). Regardless of the presence or absence of cross-linking, a horizontally stable HG of 2 mm or more was observed even after 6 months of GBR. In our previous study including the posterior region, the HGR was 71.0% for 3D-PFTM only, whereas it was 84.2% in the CCM group and 84.0% in the NCCM group, when the resorbable membrane was used together [15]. In an animal experiment by Shin et al. [36], increased HGR was observed in the Ti mesh group using the resorbable collagen membrane compared to that in the group treated with Ti mesh alone. In this study, the average HGR of 28 sites was 83.41% ± 13.40%, which is similar to that reported in previous studies. Moreover, as in previous studies, the presence or absence of cross-linking did not significantly affect the HGR value.

Jiang et al. [14] reported that the results of GBR in peri-implant bony defects in a single maxillary anterior region were affected by the location of the bony envelope, regardless of the submerged and transmucosal healing groups. In other words, immediately after implant placement, the results of bone graft overaddition to the outside of the bony envelope by −0.82 ± 0.68 mm and −0.67 ± 0.60 mm were shown in the submerged healing group and transmucosal healing group, respectively, whereas after the 6 month healing period, the horizontal volume was reduced inside the bony envelope to 0.16 ± 0.33 mm and 0.24 ± 0.46 mm, respectively. Therefore, when GBR is performed horizontally with only particulate bone grafts and resorbable membranes, regardless of how horizontal bone grafts overadd outside the bony envelope, new bone formation may show predictability only within the bony envelope. However, in this study using 3D-PFTM, the outermost position of bone grafts from the bony envelope immediately after GBR was −1.90 ± 0.88 mm on average, and overadding to the outside bony envelope was also observed. Even after the 6 month healing period, the average was −1.42 ± 0.93 mm, and it was found to maintain a stable hard tissue volume by still being located in the outside bony envelope. These results showed that, regardless of the presence or absence of cross-linking, when 3D-PFTM was used together in all cases of both groups of collagen membranes, stable HG was observed outside the bony envelope even after 6 months of healing. This excellent hard tissue volume stability is due to the fact that 3D-PFTM is firmly fixed to the implant fixture and has a stable anti-pressure effect, unlike the resorbable membrane; space making for a long time is difficult with the resorbable membrane due to the compression of the soft tissues of the maxillary anterior region, including the lips [14,15].

The results of this study should be interpreted with caution because of the relatively small number of subjects and short follow-up time. In addition, in this study, there was no control for other conditions that could affect GBR outcomes, such as soft tissue conditions including the gingival phenotype [37] and the size of bony defects around the implants. Regenerated hard tissue determined by CBCT may not be a true new bone with histological significance. Finally, since the hard tissue change was measured only two-dimensionally at the platform level of the implant fixture, future studies are needed to evaluate three-dimensional changes.

## 5. Conclusions

Despite the limitations of this study, when GBR is performed using 3D-PFTM at the same time as implant placement in dehiscence bony defects in the anterior maxilla, HG can be predicted regardless of whether the collagen membrane used together is cross-linked. In addition, 3D-PFTM may confer predictable hard tissue volume stability even after the healing period of hard tissue augmented outside the bony envelope by GBR. However, long-term results following functional loading are required before recommending this technique for daily practice, unlike the short-term follow-up period in this study.

## Figures and Tables

**Table 1:** Characteristics of subjects.

	3D-PFTM + CCM (Test)	3D-PFTM + NCCM (Control)	Total
Ns	14	14	28
Ni	14	14	28
Mean age (range)	48.2 ± 18.6	49.5 ± 19.5	48.9 ± 18.7
	(22–69)	(20–84)	(20≠84)
Sex			
Men, Ns (Ni)	7 (7)	7 (7)	14 (14)
Women, Ns (Ni)	7 (7)	7 (7)	14 (14)

N, number of subjects; Ni, number of sites (implants); 3D-PFTM, three-dimensional preformed titanium mesh; CCM, cross-linked collagen membrane; NCCM, non-cross-linked collagen membrane.

**Table 2:** Clinical evaluation of the mesh exposure, ISQ value, and survival rate of implants in both groups.

	Test	Control	Total
Mesh exposure (N)	1	1	2
Mesh exposure rate (%)	6.67	6.67	6.67
ISQ (mean ± SD)	73.77 ± 6.34	74.45 ± 7.53	74.11 ± 6.80
Survival rate of implant (%)	100	100	100

N, number; ISQ, implant stability quotient; SD, standard deviation; Test group, 3D-PFTM + cross-linked collagen membrane; Control group, 3D-PFTM + non-cross-linked collagen membrane.

**Table 3:** Radiological assessment of BA, HG, and HGR between the groups.

	Test (n = 14)	Control (n = 14)	Total (n = 28)
BA (mean ± SD) (mm)	2.91 ± 0.75	2.76 ± 0.62	2.83 ± 0.68
*p*-value	0.570		
HG (mean ± SD) (mm)	2.44 ± 0.75	2.27 ± 0.63	2.35 ± 0.68
*p*-value	0.536		
BR (mean ± SD) (mm)	0.47 ± 0.47	0.49 ± 0.40	0.48 ± 0.43
*p*-value	0.511		
HGR (mean ± SD) (%)	84.25 ± 14.19	82.56 ± 13.04	83.41 ± 13.40
*p*-value	0.482		

SD, standard deviation; BA, bone augmentation immediately after surgery; HG, hard tissue gain after 6 months of healing; BR, bone graft reduction (BA-HG); HGR, percentage of HG compared with BA (HG/BA); Test group, 3D-PFTM + cross-linked collagen membrane used; Control group, 3D-PFTM + non-cross-linked collagen membrane used.

**Table 4:** Distance from the boundary line of the bony envelope to the hard tissue profile immediately after augmentation and after 6 months of healing.

	Test (n = 14)	Control (n = 14)	Total (n = 28)
After augmentation (mean ± SD) (mm)	−2.01 ± 0.89	−1.82 ± 0.82	−1.91 ± 0.84
*p*-value		0.571	
After 6 months (mean ± SD) (mm)	−1.54 ± 1.02	−1.34 ± 0.79	−1.44 ± 0.90
*p*-value		0.566	

Test group: 3D-PFTM + cross-linked collagen membrane; Control group: 3D-PFTM + non-cross-linked collagen membrane.

## Data Availability

The datasets generated or analyzed during the current study are available from the corresponding author upon reasonable request.
